# Supplementary material for: Dominant Vibrio cholerae phage exhibits lysis inhibition sensitive to disruption by a defensive phage satellite
Source: eLife. 2020 Apr 24;9:e53200. doi: 10.7554/eLife.53200 (PMC7182436; doi:10.7554/eLife.53200)
Supplement: Supplementary file 1. — The gene products referred to in this work relate to open reading frames (ORFs) as noted in the ‘Locus Tag Note’. [file elife-53200-supp1.docx]

| **gp as Referenced in this Work** | **GenBank Reference Genome** | **Name** | **Locus Tag** | **Locus Tag Note** | **Protein ID** |
| --- | --- | --- | --- | --- | --- |
| **gp139** | MH310934.1 | NA | ICP12006E_140 | Also designated as **ORF139** | AXQ70765.1 |
| **gp138** | MH310934.1 | arrA | ICP12006E_139 | Also designated as **ORF138** | AXQ70764.1 |
| **gp137** | MH310934.1 | teaA | ICP12006E_138 | Also designated as **ORF137** | AXQ70763.1 |
| **gp136** | MH310934.1 | NA | ICP12006E_137 | Also designated as **ORF136** | AXQ70762.1 |

**Supplemental File 1. ICP1_2006_E gene product (gp) GenBank References.** The gene products referred to in this work relate to open reading frames (ORFs) as noted in the ‘Locus Tag Note’.
