# Supplementary material for: Dominant Vibrio cholerae phage exhibits lysis inhibition sensitive to disruption by a defensive phage satellite
Source: eLife. 2020 Apr 24;9:e53200. doi: 10.7554/eLife.53200 (PMC7182436; doi:10.7554/eLife.53200)
Supplement: Supplementary file 2. — BLASTP was used to find homologs that share 30% identity with TeaA over 85% of the query. The GenBank ID, description, number of transmembrane domains (TMD) as predicted by TMHMM Server 2.0, and organism is listed for each homolog. Whether or not an ArrA homolog was found in the same organism is noted in the ‘ArrA’ column. Additionally, the adjacent upstream and downstream genes were analyzed for TMDs. GenBank descriptions are color coded. Due to the number of homologs analyzed, this table is only available as a spreadsheet as Source data 1. [file elife-53200-supp2.docx]

**Supplemental File 2. TeaA homologs.** BLASTP was used to find homologs that share 30% identity with TeaA over 85% of the query. The GenBank ID, description, number of transmembrane domains (TMD) as predicted by TMHMM Server 2.0, and organism is listed for each homolog. Whether or not an ArrA homolog was found in the same organism is noted in the ‘ArrA’ column. Additionally, the adjacent upstream and downstream genes were analyzed for TMDs. GenBank descriptions are color coded. Due to the number of homologs analyzed, this table is only available as a spreadsheet as Supplemental Table Source Data – Supp Table 2 Source Data.
