# Supplementary material for: Dominant Vibrio cholerae phage exhibits lysis inhibition sensitive to disruption by a defensive phage satellite
Source: eLife. 2020 Apr 24;9:e53200. doi: 10.7554/eLife.53200 (PMC7182436; doi:10.7554/eLife.53200)
Supplement: Supplementary file 3. — BLASTP was used to find proteins with 20% identity to ArrA over 75% of the query. The GenBank ID, description, number of transmembrane domains (TMD) as predicted by TMHMM Server 2.0, and organism is listed for each homolog. Whether or not a TeaA homolog was found in the same organism is noted in the ‘TeaA’ column. Additionally, the adjacent upstream and downstream genes of each homolog were analyzed for TMDs. GenBank descriptions are color coded. The source data for this table is available in Source data 1. [file elife-53200-supp3.docx]

| **GenBank Number** | **GenBank Description** | **TMD** | **Organism** | **TeaA** | **Upstream Gene** | **TMD in Upstream Gene** | **Upstream Gene GenBank Description** | **Downstream Gene** | **TMD in Downstream Gene** | **Downstream Gene GenBank Description** |
| --- | --- | --- | --- | --- | --- | --- | --- | --- | --- | --- |
| AUR92313.1 | TMhelix containing protein | 4 | Vibrio phage 1.170.O._10N.261.52.C3 | Yes | AUR92312.1 | 1 | TMhelix containing protein | AUR92314.1 | 2 | TMhelix containing protein |
| YP_004251080.1 | hypothetical protein | 2 | Vibrio phage ICP1 | Yes | YP_004251079.1 | 2 | hypothetical protein | YP_004251081.1 | 1 | hypothetical protein |
| AUR87507.1 | TMhelix containing protein | 2 | Vibrio phage 1.101.O._10N.261.45.C6 | No | AUR87506.1 | 1 | TMhelix containing protein | AUR87508.1 | 2 | TMhelix containing protein |
| BAV80889.1 | hypothetical protein | 2 | Vibrio phage RYC | No | BAV80888.1 | 0 | hypothetical protein | BAV80890.1 | 2 | hypothetical protein |
| AUR89285.1 | TMhelix containing protein | 2 | Vibrio phage 1.121.O._10N.286.46.C4 | No | AUR89284.1 | 1 | coil containing protein | AUR89286.1 | 2 | TMhelix containing protein |
| AUR84891.1 | TMhelix containing protein | 2 | Vibrio phage 1.063.O._10N.261.45.C7 | No | AUR84890.1 | 1 | TMhelix containing protein | AUR84892.1 | 2 | TMhelix containing protein |
| AUR94238.1 | TMhelix containing protein | 4 | Vibrio phage 1.193.O._10N.286.52.C6 | Yes | AUR94237.1 | 1 | TMhelix containing protein | AUR94239.1 | 2 | TMhelix containing protein |
| YP_007877418.1 | hypothetical protein | 4 | Vibrio phage Helene 12B3 | No | YP_007877417.1 | 1 | hypothetical protein | YP_007877419.1 | 2 | hypothetical protein |
| YP_009222873.1 | hypothetical protein | 4 | Vibrio phage Eugene 12A10 | Yes | YP_009222872.1 | 1 | hypothetical protein | YP_009222874.1 | 2 | hypothetical protein |
| AUR91665.1 | TMhelix containing protein | 5 | Vibrio phage 1.161.O._10N.261.48.C5 | No | AUR91664.1 | 1 | TMhelix containing protein | AUR91666.1 | 2 | TMhelix containing protein |
| AUR93432.1 | TMhelix containing protein | 4 | Vibrio phage 1.187.O._10N.286.49.F1 | No | AUR93431.1 | 1 | coil containing protein | AUR93433.1 | 2 | TMhelix containing protein |

**Supplemental File 3. ArrA homologs.** BLASTP was used to find proteins with 20% identity to ArrA over 75% of the query. The GenBank ID, description, number of transmembrane domains (TMD) as predicted by TMHMM Server 2.0, and organism is listed for each homolog. Whether or not a TeaA homolog was found in the same organism is noted in the ‘TeaA’ column. Additionally, the adjacent upstream and downstream genes of each homolog were analyzed for TMDs. GenBank descriptions are color coded. The source data for this table is available as Supplemental Table Source Data – Supp Table 3 Source Data.
