# Supplementary material for: Dominant Vibrio cholerae phage exhibits lysis inhibition sensitive to disruption by a defensive phage satellite
Source: eLife. 2020 Apr 24;9:e53200. doi: 10.7554/eLife.53200 (PMC7182436; doi:10.7554/eLife.53200)
Supplement: Transparent reporting form [file elife-53200-transrepform.docx]

***eLife’s* transparent reporting form**

We encourage authors to provide detailed information *within their submission* to facilitate the interpretation and replication of experiments. Authors can upload supporting documentation to indicate the use of appropriate reporting guidelines for health-related research (see [EQUATOR Network](http://www.equator-network.org/%20)), life science research (see the [BioSharing Information Resource](https://biosharing.org/)), or the [ARRIVE guidelines](http://www.plosbiology.org/article/info:doi/10.1371/journal.pbio.1000412) for reporting work involving animal research. Where applicable, authors should refer to any relevant reporting standards documents in this form.

If you have any questions, please consult our Journal Policies and/or contact us: [editorial@elifesciences.org](mailto:editorial@elifesciences.org).

**Sample-size estimation**

- You should state whether an appropriate sample size was computed when the study was being designed
- You should state the statistical method of sample size computation and any required assumptions
- If no explicit power analysis was used, you should describe how you decided what sample (replicate) size (number) to use

Please outline where this information can be found within the submission (e.g., sections or figure legends), or explain why this information doesn’t apply to your submission:

No explicit power analysis was used to determine sample sizes for experiments – all experiments were repeated with a minimum of three biological replicates (e.g. three distinct bacterial colonies) as per convention. Details for the exact number of replicates can be found in the legend of each figure.

**Replicates**

- You should report how often each experiment was performed
- You should include a definition of biological versus technical replication
- The data obtained should be provided and sufficient information should be provided to indicate the number of independent biological and/or technical replicates
- If you encountered any outliers, you should describe how these were handled
- Criteria for exclusion/inclusion of data should be clearly stated
- High-throughput sequence data should be uploaded before submission, with a private link for reviewers provided (these are available from both GEO and ArrayExpress)

Please outline where this information can be found within the submission (e.g., sections or figure legends), or explain why this information doesn’t apply to your submission:

Each experiment was completed with at least 3 biological replicates (e.g. three distinct bacterial colonies) and the minimum number of replicates is available in the figure legends. We show all data points (either as points or included in the calculation of standard deviation) without removing outliers and have included the source data in additional source files. We used no high-throughput sequencing datasets.

**Statistical reporting**

- Statistical analysis methods should be described and justified
- Raw data should be presented in figures whenever informative to do so (typically when N per group is less than 10)
- For each experiment, you should identify the statistical tests used, exact values of N, definitions of center, methods of multiple test correction, and dispersion and precision measures (e.g., mean, median, SD, SEM, confidence intervals; and, for the major substantive results, a measure of effect size (e.g., Pearson's r, Cohen's d)
- Report exact p-values wherever possible alongside the summary statistics and 95% confidence intervals. These should be reported for all key questions and not only when the p-value is less than 0.05.

Please outline where this information can be found within the submission (e.g., sections or figure legends), or explain why this information doesn’t apply to your submission:

Statistical tests were completed on GraphPad PRISM and tests are specified in the legend of each figure. P-values are communicated by the number of asterisks when appropriate. We can add exact p-values to the legends upon reviewer request however we believe the ranges represented by asterisks communicates our findings and significance successfully to the reader.

(For large datasets, or papers with a very large number of statistical tests, you may upload a single table file with tests, Ns, etc., with reference to sections in the manuscript.)

**Group allocation**

- Indicate how samples were allocated into experimental groups (in the case of clinical studies, please specify allocation to treatment method); if randomization was used, please also state if restricted randomization was applied
- Indicate if masking was used during group allocation, data collection and/or data analysis

Please outline where this information can be found within the submission (e.g., sections or figure legends), or explain why this information doesn’t apply to your submission:

This work does not include classical group allocation found in clinical studies however homology was used to split the LidI homologs into two groups and identify holin and antiholin like sequences via BLASTP. Grouping of LidI homologs is described in the text and visualized in Figure 4-Figure Supplement 1. BLASTP analysis was used to identify theoretical homologs and specifications are outlined in the figure legends and materials and methods section of the text.

**Additional data files (“source data”)**

- We encourage you to upload relevant additional data files, such as numerical data that are represented as a graph in a figure, or as a summary table
- Where provided, these should be in the most useful format, and they can be uploaded as “Source data” files linked to a main figure or table
- Include model definition files including the full list of parameters used
- Include code used for data analysis (e.g., R, MatLab)
- Avoid stating that data files are “available upon request”

Please indicate the figures or tables for which source data files have been provided:

Raw data is shown in all bar graphs in addition to summary statistics and additional source datafiles are provided for all figures other than Figure 4 – Figure Supplement 3 due to our belief that the raw data within that figure will not be of use to readers outside of the qualitative conclusions reached in this work. No specialized code was used during analysis of data for this manuscript – all programs are specified in the materials and methods and should be attributed to their original creators as designated by the included references.
